# Association between physical activity and long-term prognosis in older patients with Stage B heart failure

**DOI:** 10.1016/j.ijcrp.2026.200700

**Published:** 2026-08-18

**Authors:** Hiroyuki Tsukahara, Koichiro Matsumura, Shun Morishita, Junko Morimoto, Satoshi Kurose, Shohei Hakozaki, Eijiro Yagi, Masafumi Ueno, Hidekazu Tanaka, Yutaka Kimura, Gaku Nakazawa

**Affiliations:** a Kindai University Faculty of Medicine, Sakai, Japan; b Department of Cardiology, Kindai University Faculty of Medicine, Sakai, Japan; c Morishita Heart Clinic, Kyoto, Japan; d Department of Cardiovascular Medicine, Arida City Hospital, Arida, Japan; e Faculty of Sports and Health Sciences, Osaka Sangyo University, Daito, Japan; f Health Science Center, Kansai Medical University Hospital, Hirakata, Japan

**Keywords:** Stage B heart failure, Physical activity, Physical function, Older adult

## Abstract

**Background:**

Physical activity is an important modifiable lifestyle factor associated with cardiovascular outcomes. However, its clinical significance in patients with Stage B heart failure (HF) remains unclear. This study investigated the association between physical activity and long-term outcomes and physical function in older patients with Stage B HF.

**Methods:**

This sub-analysis of the multicenter PAPRIKA-HF registry assessed physical activity using the International Physical Activity Questionnaire (IPAQ) and categorized patients into high and low physical activity groups based on their median IPAQ score. The primary outcome was a composite of all-cause death, myocardial infarction hospitalization, and HF hospitalization during 2-year follow-up.

**Results:**

Older outpatients with Stage B HF (N, 308; median age, 78 years; 45% women) were included. The median physical activity level was 693MET-min/week, and 68 patients (22%) reported no physical activity. Patients with low physical activity levels had a significantly higher composite endpoint incidence than those with high physical activity levels (10.0% [16/160 patients] vs. 2.7% [4/148 patients], log-rank test; *p* = 0.008). On multivariable Cox regression adjusted for AHEAD score, low physical activity was independently associated with an increased risk of the composite endpoint (hazard ratio, 3.70; 95% confidence interval, 1.23–11.09; *p* = 0.01). Patients with low physical activity had significantly lower skeletal muscle mass, handgrip strength, and gait speed.

**Conclusion:**

Lower physical activity was independently associated with adverse cardiovascular outcomes and impaired physical function in older patients with Stage B HF. Thus, physical activity may identify high-risk individuals before symptomatic HF develops.

## Highlights

• Daily physical activity independently predicted adverse outcomes in older Stage B HF.
• Lower physical activity was associated with impaired muscle mass, strength, and gait speed.
• Habitual physical activity may identify high-risk patients before symptomatic HF develops.
• Assessing physical activity may help guide preventive strategies in Stage B HF.

## 1 Introduction

Heart failure (HF), a major cardiovascular disease whose prevalence continues to increase worldwide with population aging, is closely associated with increased mortality and impaired quality of life [1]. Despite substantial advances in pharmacological therapy, the prognosis of patients with symptomatic HF remains poor [2]. Therefore, increasing attention has been directed toward risk assessment and preventive interventions in the earlier stages of the HF continuum.

Physical activity is an important lifestyle factor that influences the development and prognosis of cardiovascular diseases and has recently gained considerable attention in geriatric medicine [3]. Furthermore, in patients with chronic HF, lower levels of physical activity are independently associated with an increased risk of mortality and hospitalization [4]. However, most previous studies focused on patients with symptomatic (Stage C) HF, in whom reduced physical activity may simply reflect exercise limitations and reduced functional capacity resulting from the HF symptoms. Consequently, it remains unclear whether low physical activity is merely a consequence of HF or an early marker of its progression.

Stage B HF, characterized by the presence of structural heart disease in the absence of HF symptoms, is a recognized high-risk indicator of the future development of symptomatic HF and cardiovascular events [5]. This stage represents a critical window of opportunity for preventive interventions aimed at delaying HF progression. Therefore, identifying modifiable prognostic factors at this stage is clinically important. Physical activity is a potentially modifiable behavioral factor, and understanding its clinical significance in the early stages of HF may provide valuable insights into strategies to prevent disease progression. However, little is known of the association between physical activity and long-term outcomes in patients with Stage B HF. Therefore, this study aimed to investigate the association between physical activity and long-term prognosis in older patients with Stage B HF. It also examined the relationship between physical activity and physical function to better understand the clinical significance of physical activity in this population.

## 2 Methods

### 2.1 Study design and population

This was a sub-analysis of the PAPRIKA-HF registry, a multicenter cohort study conducted at four institutions in Japan between June 2021 and May 2023. A total of 331 older outpatients with Stage B HF were enrolled from four participating institutions. The detailed study methods were previously reported [6]. Eligible participants were aged ≥ 65 years, had no prior hospitalization for HF, and were diagnosed with Stage B HF based on their symptoms, medical history, medication use, and echocardiographic findings. The exclusion criteria were active malignancy, dialysis dependence, inability to undergo a physical function assessment, and use of loop diuretics, with the latter criterion applied to minimize the inclusion of patients with symptomatic HF. Participants with unavailable body composition measurements (n = 10), for whom laboratory data were missing (n = 4), or whose questionnaires were incomplete (n = 9) were also excluded from the present analysis. This study was conducted in accordance with the Declaration of Helsinki and approved by the institutional ethics committees of all participating institutions. Written informed consent was obtained from all participants.

### 2.2 Definition of stage B HF

Stage B HF was defined according to the 2022 American College of Cardiology/American Heart Association/Heart Failure Society of America guidelines as the presence of structural heart disease or cardiac dysfunction without any signs or symptoms of HF [7]. Structural heart abnormalities were identified using echocardiography according to the criteria derived from the Atherosclerosis Risk in Communities (ARIC) study and included regional wall motion abnormalities, valvular heart disease, left ventricular enlargement, left ventricular hypertrophy, systolic dysfunction, or diastolic dysfunction [8].

### 2.3 Data collection

The participants' baseline demographic and clinical characteristics, including age, sex, body mass index, comorbidities (hypertension, dyslipidemia, diabetes mellitus, atrial fibrillation, prior myocardial infarction, and prior cardiac surgery), and medication use were collected at enrollment. The laboratory data and echocardiographic parameters obtained at baseline were also recorded.

### 2.4 Assessment of physical activity

Habitual physical activity was assessed using the interviewer-administered short form of the International Physical Activity Questionnaire (IPAQ; short form), which evaluates physical activity performed during the preceding 7 days and categorizes activities into walking, moderate-intensity activity, and vigorous-intensity activity [9]. Total physical activity was calculated as metabolic equivalents in minutes per week (METs-min/week) using the standard IPAQ scoring protocol: walking MET-min/week = walking min/day × days/week × 3.3 METs; moderate activity METs-min/week = moderate activity min/day × days/week × 4.0 METs; and vigorous activity METs-min/week = vigorous activity min/day × days/week × 8.0 METs. Total physical activity was calculated as the sum of the three components.

### 2.5 Assessment of physical function

Each participant's physical function was assessed at baseline. Skeletal muscle mass was measured using bioelectrical impedance analysis, while appendicular skeletal muscle mass index (SMI) was calculated. Handgrip strength was measured twice for each hand, and the highest value was included in the analysis [10]. Gait speed was assessed as the usual walking speed over a 4- or 6-m walking course [10].

### 2.6 Study outcomes

Patients were divided into high and low physical activity groups according to the median total physical activity value assessed using the IPAQ. As no established IPAQ cutoff has been reported in older patients with Stage B HF, the participants were categorized according to the cohort median value. The primary outcome was the composite endpoint of all-cause mortality, hospitalization for myocardial infarction, and hospitalization for HF during the 2-year follow-up period. Secondary outcomes included all-cause mortality and hospitalization for myocardial infarction and HF. Physical function parameters, including SMI, handgrip strength, and gait speed, were compared between the two groups. Clinical events were confirmed through a medical record review, follow-up questionnaires sent to referral hospitals, and telephone interviews when necessary.

### 2.7 Statistical analyses

Continuous variables are presented as median (interquartile range) and were compared using the Mann–Whitney *U* test. Categorical variables are presented as number (percentage) and were compared using the χ^2^ test. Comparisons of physical function measures between the two IPAQ groups, performed separately in men and women, were considered exploratory; therefore, no adjustment for multiple comparisons was applied. Cumulative event rates were estimated using the Kaplan–Meier method and compared using the log-rank test. Cox proportional hazard models were used to evaluate the association between low physical activity and the primary outcome. A multivariate adjustment was performed using the AHEAD score, a prognostic risk score for patients with HF [11]. For the sensitivity analyses, additional Cox proportional hazards models were constructed with further adjustments for female sex, high-sensitivity C-reactive protein (hsCRP) level, and B-type natriuretic peptide (BNP) level. Furthermore, total physical activity was analyzed as a continuous variable, and hazard ratios (HRs) are expressed per 500 METs-min/week increase. Statistical analyses were performed using JMP Student Edition 19.0.0 (SAS Institute Inc., Cary, NC, USA), which provides the full statistical functionality of JMP and JMP Pro. Statistical significance was defined as a two-sided value of *p* < 0.05.

## 3 Results

### 3.1 Patient characteristics

**Fig. 1:**
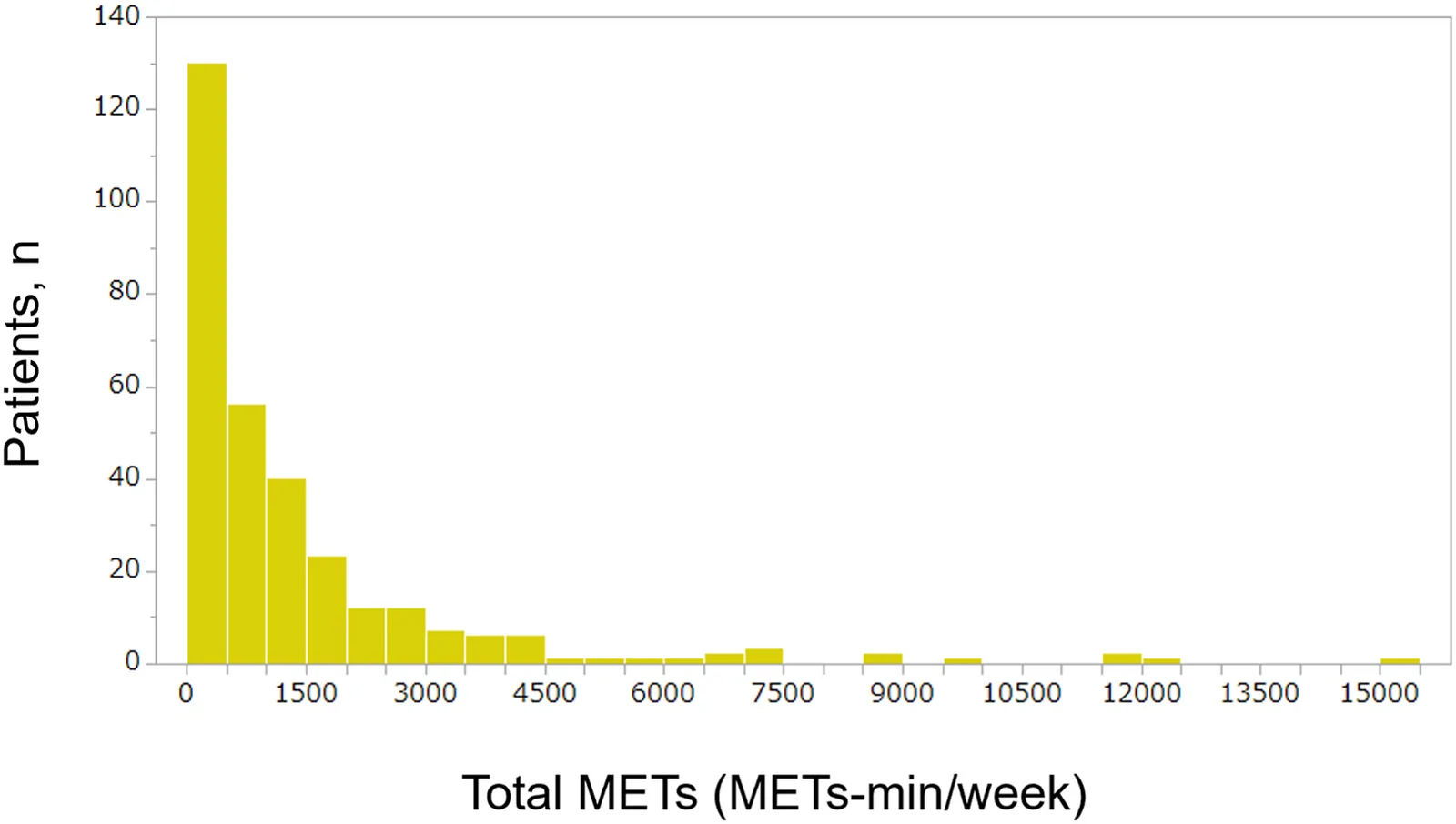
**Distribution of total METs-min/week**METs-min/week, metabolic equivalents in minutes per week.

A total of 308 patients (median age, 78 years; 45% female) were included in the analysis (Supplemental Fig. 1). The distribution of total physical activity assessed using the IPAQ is shown in Fig. 1. The median total physical activity level was 693 METs-min/week, and the patients were categorized into a high IPAQ group (n = 148) and a low IPAQ group (n = 160) using the median value as the cutoff. The components of the IPAQ according to physical activity group are presented in Supplemental Table 1. Patients in the high IPAQ group performed substantially more walking activity and slightly more moderate-intensity activity than those in the low IPAQ group, whereas vigorous-intensity activity was negligible in both groups. In the study population, 68 patients (22%) reported no physical activity (0 MET-min/week).

The participants' baseline characteristics are shown in Table 1. Patients in the low IPAQ group were older and more frequently female than those in the high IPAQ group. In contrast, dyslipidemia, prior myocardial infarction, and antiplatelet use were more prevalent in the high IPAQ group.

**Table 1:** Patients’ background characteristics.

	Low IPAQ Soft-enter Run-on = 160)	High IPAQ Soft-enter Run-on = 148)	*p* value
Age (years)	80 (74–84)	78 (73–82)	0.02
Female	89 (56)	51 (34)	< 0.001
Body mass index (kg/m^2^)	22.8 (20.9–25.2)	23.3 (21.3–25.4)	0.34
Comorbidity			
Hypertension	130 (81)	129 (87)	0.16
Dyslipidemia	99 (62)	115 (78)	< 0.01
Diabetes mellitus	44 (28)	46 (31)	0.49
Atrial fibrillation	62 (39)	45 (30)	0.12
Prior myocardial infarction	20 (13)	44 (30)	< 0.001
Prior cardiac surgery	45 (28)	30 (20)	0.11
Prescription			
ACEi/ARB/ARNI	92 (58)	99 (67)	0.09
Beta blockers	75 (47)	80 (54)	0.21
SGLT2i	12 (8)	16 (11)	0.31
Antiplatelets	71 (44)	83 (56)	0.04
Anticoagulants	54 (34)	51 (34)	0.90

Data are presented as median (25th to 75th percentile) or number (%).

ACEi, angiotensin-converting enzyme inhibitor; ARB, angiotensin II receptor blocker; ARNI, angiotensin receptor-neprilysin inhibitor; IPAQ, International Physical Activity Questionnaire; SGLT2i, sodium glucose cotransporter 2 inhibitor.

### 3.2 Laboratory and echocardiographic findings

Patients in the low IPAQ group had lower hemoglobin levels and higher hsCRP and BNP levels than those in the high IPAQ group (Table 2). No significant intergroup differences were observed in left atrial diameter, left ventricular end-diastolic diameter, left ventricular ejection fraction, or E/e'.

**Table 2:** Laboratory and echocardiographic data.

	Low IPAQ Soft-enter Run-on = 160)	High IPAQ Soft-enter Run-on = 148)	*p* value
Laboratory			
Hemoglobin (g/dL)	12.8 (11.5–13.9)	13.4 (12.2–14.6)	< 0.01
Serum creatinine (mg/dL)	0.9 (0.7–1.1)	0.9 (0.8–1.1)	0.06
Serum albumin (g/dL)	4.1 (3.9–4.3)	4.2 (4.0–4.4)	0.15
Uric acid (mg/dL)	5.6 (4.6–6.5)	5.4 (4.7–6.2)	0.55
HsCRP (mg/dL)	0.10 (0.05–0.28)	0.05 (0.04–0.12)	0.001
BNP (pg/mL)	80 (38–146)	52 (24–99)	< 0.001
Echocardiography			
Left atrial diameter (mm)	40 (34–44)	39 (34–43)	0.63
Left ventricular end-diastolic diameter (mm)	44 (41–48)	45 (42–49)	0.11
Left ventricular ejection fraction (%)	67 (60–71)	65 (59–70)	0.25
E/eʹ	11.0 (9.0–14.0)	11.0 (8.9–13.1)	0.83

Data are presented as median (25th to 75th percentile) or number (%).

BNP, B-type natriuretic peptide; hsCRP, high-sensitivity C-reactive protein; IPAQ, International Physical Activity Questionnaire.

### 3.3 Association between physical activity and physical function

**Fig. 2:**
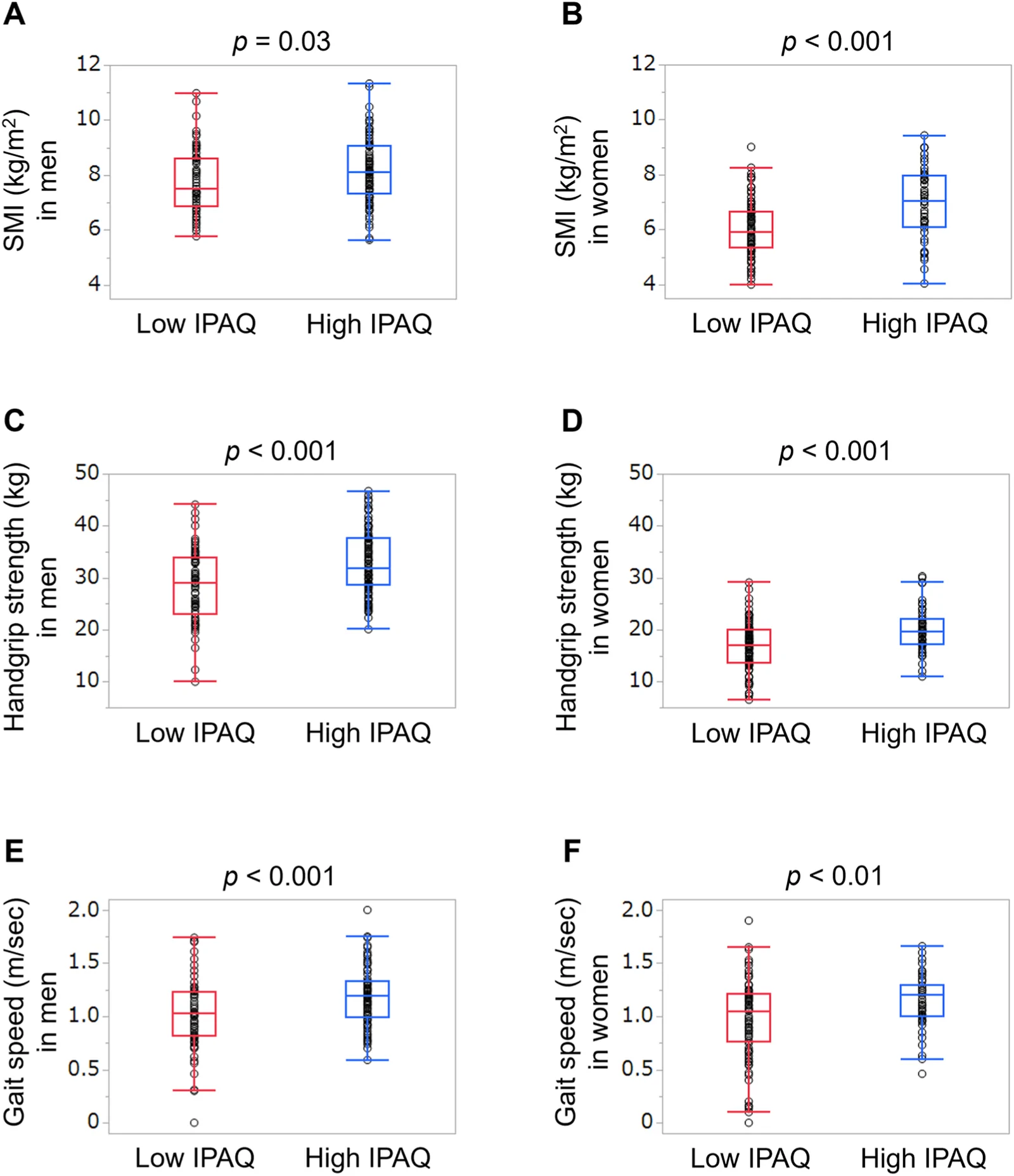
**Physical function according to physical activity status** (A) SMI in men; (B) SMI in women; (C) handgrip strength in men; (D) handgrip strength in women; (E) gait speed in men; (F) gait speed in women IPAQ, International Physical Activity Questionnaire; SMI, skeletal muscle mass index.

Fig. 2 compares physical function according to physical activity status. The men in the low IPAQ group had a significantly lower appendicular SMI (7.5 [6.9–8.6] vs. 8.1 [7.3–9.1] kg/m^2^, *p* = 0.03), handgrip strength (29.1 [23.0–33.8] vs. 31.8 [28.7–37.7] kg, *p* < 0.001), and gait speed (1.0 [0.8–1.2] vs. 1.2 [1.0–1.3] m/s, *p* < 0.001) than those in the high IPAQ group. Similarly, the women in the low IPAQ group had a significantly lower appendicular SMI (5.9 [5.4–6.6] vs. 7.0 [6.1–8.0] kg/m^2^, *p* < 0.001), handgrip strength (17.0 [13.6–20.0] vs. 19.7 [17.1–22.0] kg, *p* < 0.001), and gait speed (1.1 [0.8–1.2] vs. 1.2 [1.0–1.3] m/s, *p* < 0.01) than those in the high IPAQ group.

### 3.4 Primary outcome

**Fig. 3:**
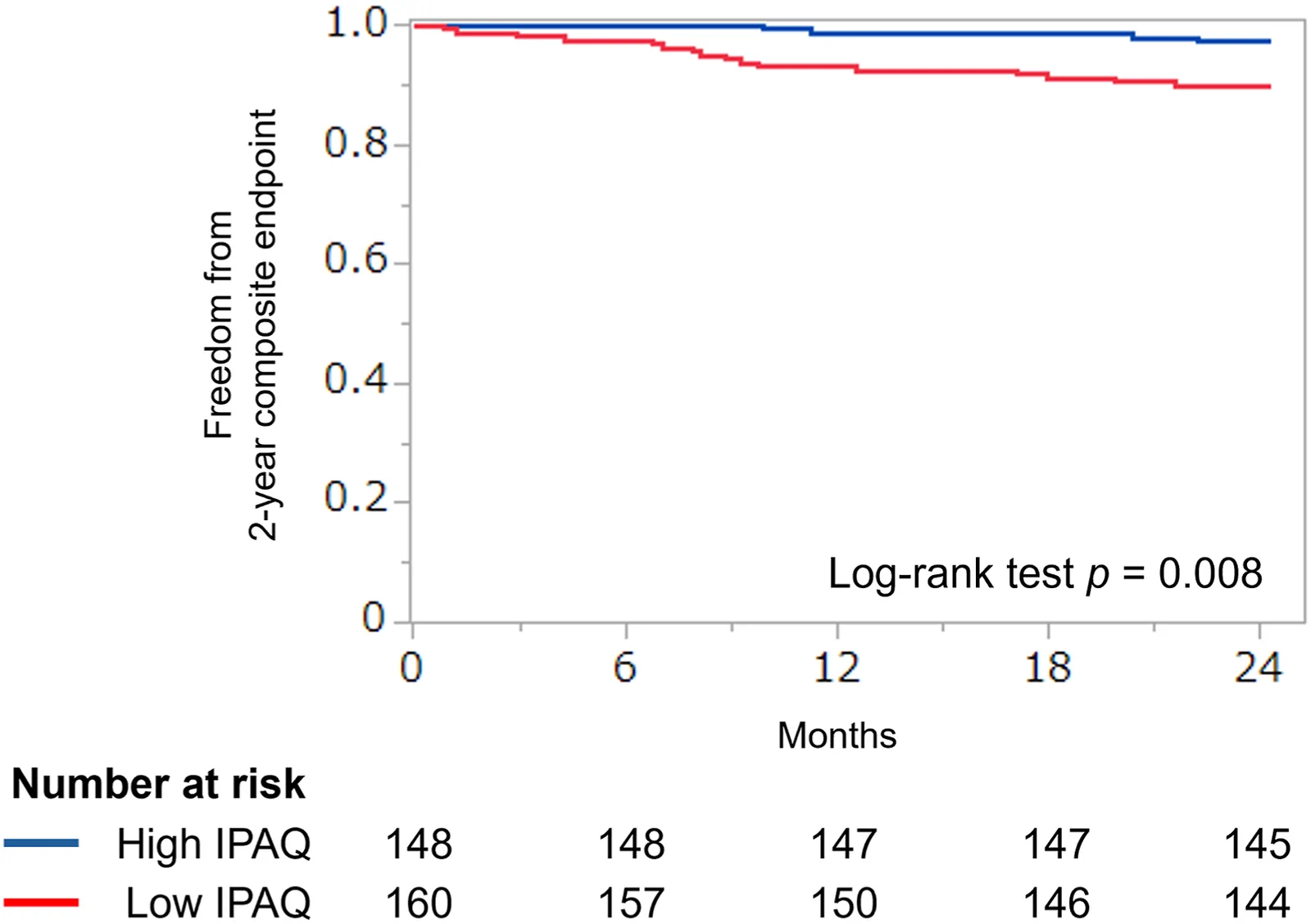
**Kaplan–Meier analyses of 2-year composite endpoint** IPAQ, International Physical Activity Questionnaire.

During the 2-year follow-up period, 20 composite events occurred, including 10 all-cause deaths, three myocardial infarctions, and seven hospitalizations for HF. A Kaplan–Meier analysis demonstrated that patients with a low IPAQ score had a significantly higher incidence of the composite endpoint than those with a high IPAQ score (10.0% vs. 2.7%, respectively, log-rank test; *p* = 0.008) (Fig. 3).

### 3.5 Secondary outcome

Regarding secondary outcomes, patients in the low IPAQ group tended to experience higher rates of all-cause death, acute myocardial infarction, and hospitalization for HF than those in the high IPAQ group, although the intergroup differences were not statistically significant (Supplemental Table 2).

### 3.6 Cox proportional hazards analyses

In multivariable Cox proportional hazards analyses, a low IPAQ score remained independently associated with an increased risk of the composite endpoint after the adjustment for AHEAD score (HR 3.70, 95% CI 1.23–11.09, *p* = 0.01). In sensitivity analysis, this association remained significant after further adjustment for sex and hsCRP and BNP levels (Table 3). In an additional analysis treating physical activity as a continuous variable, higher physical activity levels were independently associated with a lower risk of the 2-year composite endpoint after adjustment for the AHEAD score (HR, 0.81 per 500 METs-min/week increase; 95% CI, 0.60–0.99; *p* = 0.04) (Table 4).

**Table 3:** Association between low physical activity and 2-year composite endpoint on multivariable Cox proportional hazards models.

	Model 1		Model 2		Model 3		Model 4	
	HR (95% CI)	*p* value	HR (95% CI)	*p* value	HR (95% CI)	*p* value	HR (95% CI)	*p* value
Low IPAQ	3.70 (1.23–11.09)	0.01	3.33 (1.09–10.17)	0.03	3.67 (1.22–11.04)	0.02	3.71 (1.22–11.31)	0.02

Model 1: AHEAD score.

Model 2: AHEAD score + female sex.

Model 3: AHEAD score + hsCRP (mg/dL).

Model 4: AHEAD score + BNP (pg/mL).

HRs represent the risk associated with a low versus high IPAQ score.

BNP, B-type natriuretic peptide; CI, confidence interval; HR, hazard ratio; hsCRP, high-sensitivity C-reactive protein; IPAQ, International Physical Activity Questionnaire.

**Table 4:** Results of multivariate Cox proportional hazards model evaluation of association between physical activity (per 500 METs-min/week increase) and 2-year composite endpoint.

	HR (95% CI)	*p* value
Physical activity (per 500 METs-min/week increase)	0.81 (0.60–0.99)	0.04

The value was adjusted for the AHEAD score.

CI, confidence interval; HR, hazard ratio; METs-min/week, metabolic equivalents in minutes per week.

## 4 Discussion

The present study investigated the association among habitual physical activity, long-term outcomes, and physical function in older patients with Stage B HF. Its major findings are as follows. First, lower physical activity was independently associated with a higher risk of the 2-year composite endpoint. Second, lower physical activity was associated with reduced skeletal muscle mass, lower handgrip strength, and slower gait speed. These findings suggest that reduced physical activity is not merely a lifestyle characteristic but may also reflect a high-risk condition accompanied by physical dysfunction. Furthermore, habitual physical activity may be a useful marker for identifying individuals at an increased risk of future cardiovascular events before symptomatic HF develops.

The association between physical activity and clinical outcomes was previously reported in both the general population and patients with cardiovascular disease [12,13]. In a large accelerometer-based meta-analysis, higher physical activity levels were associated with lower all-cause mortality, whereas greater sedentary time was associated with increased mortality risk [12]. Physical activity is a recognized modifiable factor that may delay frailty progression. Among male survivors of myocardial infarction, maintaining high levels of physical activity or increasing physical activity after myocardial infarction is associated with a lower risk of mortality [13]. However, the clinical significance of physical activity in patients with Stage B HF has not been adequately investigated. Although patients with prior myocardial infarction may represent a subset of those with Stage B HF, the concept of Stage B HF encompasses a broader spectrum of structural heart diseases, including left ventricular hypertrophy, ventricular enlargement, valvular heart disease, and diastolic dysfunction. Therefore, it remains unclear whether the findings observed in patients with coronary artery disease can be generalized to older patients with Stage B HF. In our study, lower physical activity levels were independently associated with impaired physical function and adverse long-term outcomes in older patients with Stage B HF. Importantly, this association remained significant after the adjustment for AHEAD score, inflammatory markers, and HF-related biomarkers. Regarding the individual components of the AHEAD score, patients in the low IPAQ group were older and had lower hemoglobin levels, whereas the prevalence of atrial fibrillation and diabetes mellitus and renal function were similar between the groups. These findings suggest that physical activity is not merely a lifestyle characteristic but may serve as an important clinical marker of physical dysfunction and increased cardiovascular risk before symptomatic HF develops. It should be noted that all-cause death accounted for half of the composite events (10 of 20 events) and may therefore have substantially contributed to the observed association with the primary composite endpoint. Although all-cause death, myocardial infarction, and HF hospitalization were each numerically more frequent in the low IPAQ group, the small number of individual events precluded reliable assessment of the association between physical activity and each component separately.

In our study, patients with lower physical activity levels had significantly lower skeletal muscle mass, handgrip strength, and gait speed. These findings suggest that reduced physical activity is closely associated with sarcopenia and physical frailty, both of which are important prognostic factors in older patients with HF [14,15]. Therefore, physical dysfunction may have partially mediated the association between lower physical activity and adverse clinical outcomes. We also observed significantly higher hsCRP levels in patients with lower physical activity levels. Chronic inflammation promotes skeletal muscle catabolism and contributes to endothelial dysfunction and atherosclerotic progression [16,17], suggesting another potential pathway linking reduced physical activity to adverse cardiovascular outcomes. However, the association between physical activity and prognosis remained significant after the adjustment for hsCRP. Furthermore, patients with lower physical activity levels had significantly higher BNP levels despite having similar echocardiographic parameters, including left ventricular ejection fraction and E/e′. These findings suggest that reduced physical activity may reflect subclinical HF-related pathophysiology not fully captured by conventional echocardiographic assessments. However, this finding also raises the possibility of reverse causation; patients with greater subclinical disease burden may have been less physically active as a consequence of their underlying condition, rather than lower physical activity itself contributing to subsequent adverse events. Therefore, residual confounding related to underlying disease severity cannot be excluded, despite adjustment for BNP and other prognostic factors. Habitual physical activity reflects multiple aspects of health status, including cardiac function, skeletal muscle function, comorbidities, and overall biological vulnerability. Taken together, our findings suggest that physical activity may serve as an integrated marker associated with long-term cardiovascular outcomes in older patients with Stage B HF.

Our findings highlight the clinical utility of assessing physical activity in routine outpatient practice. The IPAQ is a simple and practical questionnaire that does not require specialized equipment or expertise, making its use feasible in daily clinical settings. However, the original IPAQ was primarily developed and validated in adults younger than 65 years, warranting careful interpretation in older populations. Although an elderly-specific version of the IPAQ (IPAQ-E) has been developed [18], Tomioka et al. demonstrated acceptable reliability and validity of the original IPAQ among community-dwelling Japanese adults aged ≥65 years [19]. The median total physical activity level of 693 MET-min/week used to categorize participants into high and low physical activity groups was cohort-specific and substantially lower than the conventional IPAQ criterion for a high physical activity level (>3000 MET-min/week), likely reflecting the advanced age and relatively low physical activity level of the present population. Thus, the terms “high” and “low” IPAQ groups should be interpreted as relative classifications within this cohort rather than conventional IPAQ activity categories, and the cutoff value may not be directly applicable to other populations. Importantly, the association with the composite endpoint was also observed when physical activity was analyzed as a continuous variable, suggesting that the findings were not solely dependent on the median cutoff. Given its simplicity and the observed association between lower physical activity and adverse outcomes in our study, the assessment of habitual physical activity using the IPAQ may serve as a useful screening tool for identifying high-risk older patients with Stage B HF. Notably, approximately 22% of the patients in the present study reported zero physical activity (0 MET-min/week), indicating that a substantial proportion of older patients with Stage B HF remain physically inactive and have considerable room for improvement. Unlike age or comorbidities, physical activity is a potentially modifiable factor that may be an attractive target for lifestyle interventions aimed at preventing HF progression. Furthermore, lower physical activity was associated with reduced skeletal muscle mass, lower handgrip strength, and slower gait speed. These findings suggest that interventions targeting physical activity should not be limited to aerobic exercise alone but may also benefit from incorporating resistance training and strategies aimed at preventing frailty. Future prospective interventional studies are warranted to determine whether increasing physical activity can prevent the progression to symptomatic HF and reduce subsequent cardiovascular events in older patients with Stage B HF.

### 4.1 Strengths and limitations

Our study has several strengths. To our knowledge, it is among the first to investigate the association between habitual physical activity and long-term outcomes in older patients with Stage B HF, the pre-symptomatic phase of HF. Another strength of this study is the combination of IPAQ-based assessment of habitual physical activity with objective assessments of physical function and body composition, including handgrip strength, gait speed, and skeletal muscle mass. This comprehensive assessment enabled us to examine the relationship between self-reported physical activity and objectively assessed physical function and body composition.

This study had several limitations. First, because of the observational nature of this study, causality between reduced physical activity and adverse outcomes could not be established, and reverse causation cannot be excluded. Patients with greater subclinical disease burden may have been less physically active because of their underlying condition, which could have contributed to the observed association between lower physical activity and adverse outcomes. Second, although the IPAQ has demonstrated acceptable reliability and validity in community-dwelling older Japanese adults, its accuracy may be limited in very old or frail individuals because of recall difficulties and potential misclassification of activity duration and intensity [19]. Therefore, measurement error, including possible overestimation of physical activity, cannot be excluded. Objective measures such as accelerometer-based assessments were not available. Third, this study included a relatively small number of composite events (n = 20), resulting in limited statistical power and a wide confidence interval for the estimated hazard ratio. Therefore, the magnitude of the observed association remains uncertain and should be interpreted with caution. Larger studies with a greater number of events are needed to confirm our findings and provide more precise estimates. Moreover, the AHEAD score, which was originally developed to predict the short- and long-term prognosis of patients with established HF, has not been specifically validated for patients with Stage B HF; therefore, residual confounding may remain despite adjustment for this score. Fourth, physical activity was assessed only at baseline, while changes in physical activity during follow-up were not evaluated. Finally, all participating institutions were located in Japan, and the study population consisted exclusively of older Japanese patients. Because physical activity patterns may be influenced by cultural and lifestyle factors, the generalizability of our findings to non-Japanese populations may be limited.

## 5 Conclusion

In older patients with Stage B HF, a lower physical activity level was independently associated with a higher risk of adverse cardiovascular outcomes and impaired physical function. These findings suggest that the assessment of habitual physical activity may be useful for identifying individuals at high risk of developing symptomatic HF.

## CRediT authorship contribution statement

**Hiroyuki Tsukahara:** Data curation, Formal analysis, Methodology, Writing – original draft. **Koichiro Matsumura:** Conceptualization, Funding acquisition, Methodology, Visualization, Writing – original draft. **Shun Morishita:** Data curation, Formal analysis, Investigation, Methodology. **Junko Morimoto:** Data curation, Formal analysis, Methodology. **Satoshi Kurose:** Conceptualization, Data curation, Formal analysis, Methodology. **Shohei Hakozaki:** Conceptualization, Data curation, Formal analysis, Methodology. **Eijiro Yagi:** Data curation, Formal analysis, Investigation, Methodology. **Masafumi Ueno:** Formal analysis, Investigation, Methodology, Supervision. **Hidekazu Tanaka:** Conceptualization, Formal analysis, Methodology, Supervision, Writing – original draft. **Yutaka Kimura:** Conceptualization, Supervision, Visualization, Writing – original draft. **Gaku Nakazawa:** Conceptualization, Supervision, Visualization, Writing – review & editing.

## Data availability statement

The data supporting the findings of this study are available from the corresponding author upon request.

## Funding statement

This study was supported by a research grant from the Sugiura 10.13039/100028926Memorial Foundation.
