# Supplementary material for: Association between physical activity and long-term prognosis in older patients with Stage B heart failure
Source: Int J Cardiol Cardiovasc Risk Prev. 2026 Aug 18;30:200700. doi: 10.1016/j.ijcrp.2026.200700 (PMC13521168; doi:10.1016/j.ijcrp.2026.200700)
Supplement: Supplementary file 1 [file mmc1.docx]

Association between physical activity and long-term prognosis in older patients with Stage B heart failure

Supplementary materials

**Supplemental Table 1.** Physical activity–related IPAQ components

|  | **Low IPAQ score  (n = 160)** | **High IPAQ score  (n = 148)** |
| --- | --- | --- |
| Walking (METs-min/week) | 66 (0–347) | 1,188 (796–1,980) |
| Moderate (METs-min/week) | 0 (0–0) | 32 (0–720) |
| Vigorous (METs-min/week) | 0 (0–0) | 0 (0–0) |
| Total (METs-min/week) | 99 (0–414) | 1681 (1,189–2,902) |

Data are presented as median (25th–75th percentile).

IPAQ, International Physical Activity Questionnaire; METs-min/week, metabolic equivalents in minutes per week

**Supplemental Table 2.** Clinical event incidence

|  | **Low IPAQ score  (n = 160)** | **High IPAQ score (n = 148)** | **Log-rank test  *p* value** |
| --- | --- | --- | --- |
| All-cause death | 7 (4.4) | 3 (2.0) | 0.22 |
| Acute myocardial infarction | 3 (1.9) | 0 (0.0) | 0.08 |
| Heart failure hospitalization | 6 (3.8) | 1 (0.7) | 0.06 |

Data are presented as n (%).

**Supplemental Figure 1.** Study flowchart

BIA, bioelectrical impedance analysis; HF; Heart Failure, IPAQ; International Physical Activity Questionnaire.
